# Biofilm formation on different dental restorative materials in the oral cavity

**DOI:** 10.1186/s12903-020-01147-x

**Published:** 2020-06-03

**Authors:** Alexander-Simon Engel, Hagen Tizian Kranz, Marvin Schneider, Jan Peter Tietze, Andree Piwowarcyk, Thorsten Kuzius, Wolfgang Arnold, Ella A. Naumova

**Affiliations:** 1 Department of Biological and Material Sciences in Dentistry, Faculty of Health, Witten/Herdecke University, Alfred Herrhausenstrasse 44, 58455 Witten, Germany; 2 Department of Prosthodontics and Dental Technology, Faculty of Health, Witten/Herdecke University, Alfred Herrhausenstrasse 44, 58455 Witten, Germany; 3 Institute for Hygiene, Faculty of Medicine, Westfälische Wilhelms-Universität Muenster, Robert Koch Strasse 41, 48149 Muenster, Germany

**Keywords:** Biofilm, Oral biofilm, Dental restoratives, Surface properties

## Abstract

**Background:**

Bacterial biofilms adhere to all tissues and surfaces in the oral cavity. Oral biofilms are responsible for the decay of human dental structures and the inflammatory degeneration of the alveolar bone. Moreover, oral biofilms on artificial materials influence the lifespan of dental prostheses and restoratives.

**Methods:**

To investigate in vivo oral biofilm formation and growth, five different dental restorative materials were analyzed and compared to human enamel. The roughness of the materials and the human enamel control probe were measured at the start of the study. The dental restorative materials and the human enamel control probe were placed in dental splints and worn for 3 h, 24 h and 72 h.

**Results:**

Scanning electron microscopy (SEM) revealed major differences between oral biofilm formation and growth on the materials compared to those on human enamel. Microbiological analyses showed that bacterial strains differed between the materials. Significant differences were observed in the roughness of the dental materials.

**Conclusions:**

It can be concluded that material roughness affects biofilm formation on dental surfaces and restoratives, but other factors, such as surface charge, surface energy and material composition, may also have an influence.

## Background

Bacterial biofilms are the main causes of pathogenic processes in the oral environment [1]. They adhere to oral surfaces, natural as well as artificial surfaces, and are responsible for cariogenic action that leads to dental decay and severely limits the lifespan of dental prostheses and restoratives [2]. Oral biofilms can further affect the tissues surrounding the tooth where they cause inflammatory processes of the gingiva, and when persistent, damage to the alveolar process can, in the worst case, result in tooth loss [3].

Biofilms are conglomerates of microorganisms (bacteria, algae & fungi) that adhere to biological and nonbiological surfaces and are functionally organized in layers [4]. For biofilm formation, microorganisms generally require a humid environment to resist dehydration [5]. Microorganisms organize into biofilms to protect against external stress in a specific environment [6]. The life processes that occur in a biofilm are severely different from those that occur in the planktonic state [7, 8]. In addition to mechanical stability, biofilm formation stimulates synergistic interactions, ensures survival in periods of starvation and prevents the displacement of extracellular enzymes [9]. In this sense, biofilms are not just conglomerates of microorganisms but are rather well-organized matrix systems. Individual prokaryotes communicate within the system through signal transduction, which modulates gene expression. They also undertake different tasks to ensure survival [10, 11]. One major task fulfilled by biofilms is the secretion of an extracellular polymeric substance (EPS) to protect the system from outside influences [12].

Different in vitro studies have demonstrated the influences of surface factors on the binding force between the underlying material and the biofilm. Generally, the negatively charged bacterial cell membrane is more prone to adhere to positively charged surfaces than to negatively charged or uncharged surfaces [13, 14]; hence, surface charge has a sizeable influence on biofilm formation. Furthermore, biofilm formation is dependent on surface energy. Bacterial adhesion is more potent on hydrophilic surfaces than on hydrophobic surfaces [15]. Finally, the roughness and topography of a surface influence the extent of bacterial adhesion on the material surface [15]. Consequently, increased surface roughness promotes bacterial adherence, and the surface roughness of the 3-dimensional topographical pattern, as well as the chemical and mechanical characteristics of each particular surface, has an impact on the oral biofilm formation rate [15, 16].

Regarding the first colonizers on a rinsed enamel surface, oral streptococci (especially *Streptococcus mutans*) are the most prominent bacteria [8, 17–20]. After inevitable pellicle formation, streptococci bind to the exposed proteins on the enamel surface. At physiological pH, pellicle proteins in the oral cavity are negatively charged [18]. Since bacteria are also negatively charged at their outer membrane, this would generally cause problems for the adherence of bacteria to pellicles [15]. Streptococci use a two-stage process to bypass this issue. First, through Ca^2+^ exposure on the bacterial surface, bridging to the pellicle proteins is directly enabled [15]. Second, Streptococci produce insoluble glucans (dextran) and produce acids by enzymatic metabolic processes. Acids lower the pH of the surrounding bacteria and alter the pH level-dependent charge of dextran [13]. The more positively charged dextran is now able to bind to negatively charged pellicle proteins.

The aim of this study was to compare biofilm adhesion and formation on different dental restorative materials with those on human enamel to detect differences in bacterial composition, growth rate, and morphology of the formed oral biofilms, all in vivo.

## Methods

### Ethical approval

Prior to the study, ethical approval was obtained from the ethics committee of Witten/Herdecke University (# 15/2016). Three volunteers (dental students, 20–25 years old) gave informed written and verbal consent to wear splints during the experimental time according to the study design. The volunteers were free of any oral and systemic diseases. For the teeth that were used to prepare the enamel discs, approval of the ethics committee of Witten/Herdecke University was also obtained (# 16/2013), and the donors gave their written informed consent. Ten extracted caries-free third molars from patients between 18 and 30 years of age were used.

### Materials

Five test materials from three different categories of dental restorative materials, composite, dental ceramics and metal alloy, were used for this study. All materials are summarized in Table 1. The teeth were stored in 0.9% NaCl containing 0.1% thymol until use for disc preparation.

**Table 1:** Summary of used materials

Materials names	Manufacturer	Composition	Code
Ceram X	Dentsply-Sirona, Konstanz, Germany	Composite	Material 1
IPS e.max	Ivoclar Vivadent, Schaan, Lichtenstein	Ceramics	Material 2
Lava Plus	3 M, Neuss, Germany	Ceramics	Material 3
Vita Enamic	Vita, Bad Säckingen, Germany	Ceramics	Material 4
CoCrMo	AmannGirbach, Koblach, Austria	Metal alloy	Material 5
Enamel		Hydroxyapatite	Control

### Study design

**Fig. 1:**
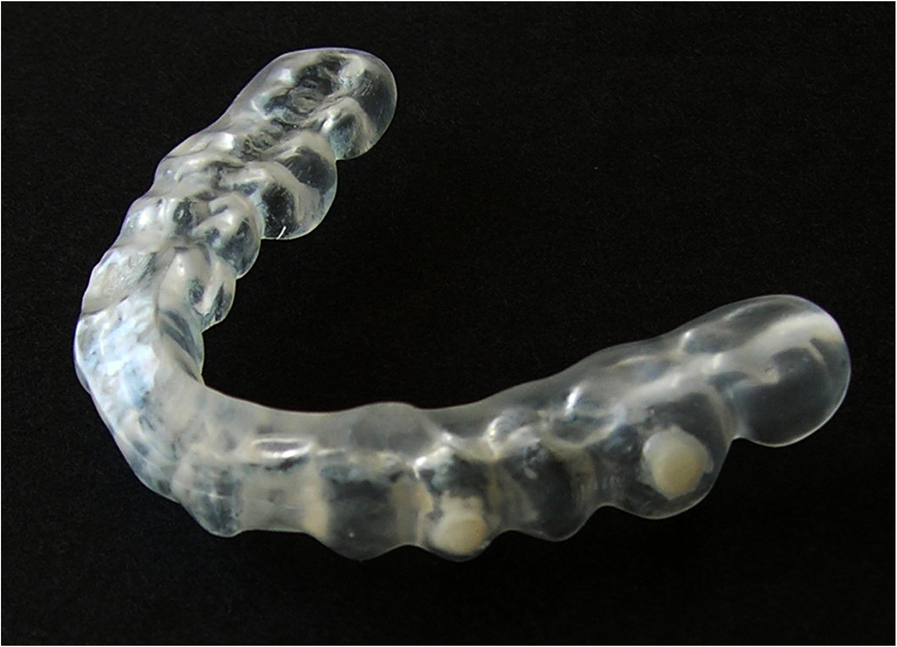
Photograph of the bite splint with fixed experimental material

**Fig. 2:**
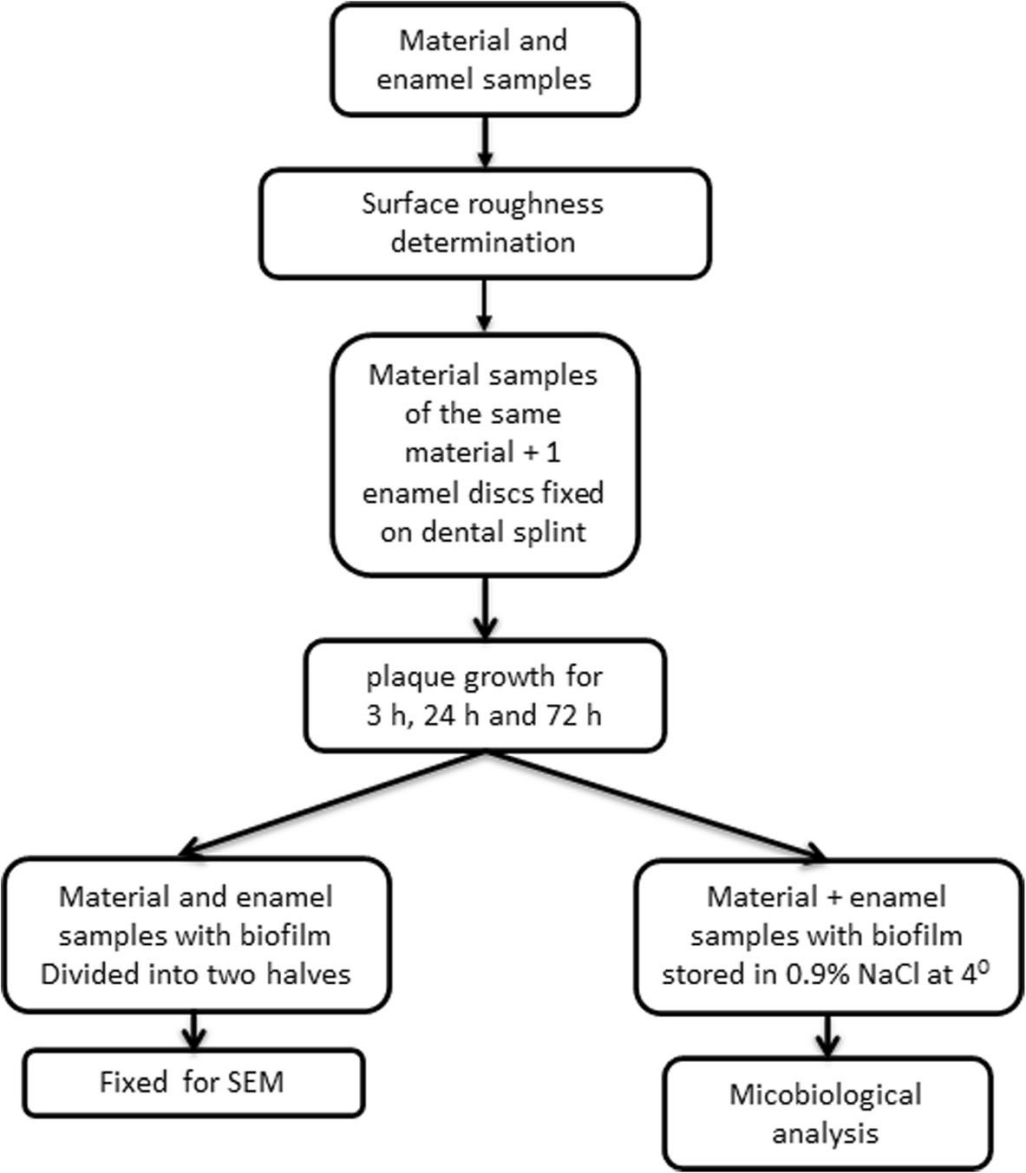
Graphic depiction of the experimental procedure

For the experiments, fifteen discs 3 mm in diameter and 2 mm thick were manufactured from each material. Human enamel discs of the same size were prepared as controls. The enamel discs underwent plasma sterilization prior to investigation [21]. Five material discs of the same material and one human enamel disc were installed in a lower jaw bite splint (Fig. 1) and worn by the volunteers to allow biofilm formation on the surface. Each volunteer was assigned to one material. The discs were located on the buccal side of the second premolar, the first molar and the second molar in both lower jaw quadrants. The experiments were repeated for each material with the identical bite splint. After each time period, the material and the enamel discs were removed, and another set was mounted on the bite splint. Prior to changing the material, the bite splints were disinfected according to standard disinfection procedures for bite splints. The volunteers were assigned to different materials, and the experiments were carried out with the same material. As only three volunteers were available, but five materials were investigated, two of the volunteers repeated the experiments with another material. To follow up on time-dependent differences in biofilm formation, the experiments were carried out for 3 h, 24 h and 72 h time periods. From every bite splint, two material discs were used for SEM analysis, two material discs were stored in 0.9% NaCl for microbiological analysis and one material disc was frozen (− 20 °C) as a backup specimen. The study protocol is depicted in detail in Fig. 2.

### Disc preparation

For preparation of the discs for material 1, rectangular wax molds were formed. The material was polymerized in the wax mold, resulting in cubic blocks from which the discs were cut. For cutting, a trepan burr (Hager & Meisinger GmbH, Neuss, Germany) was used with a speed of 10.000 rpm under water cooling. Finally, the surface was polished according to the manufacturer’s instructions.

Material 2 was delivered by the manufacturer as prefabricated bars with a diameter of 3 mm. From these bars, discs were cut using a cutoff wheel at a speed of 15.000 rpm under water cooling. The surface of the discs was polished according to the manufacturer’s instructions using Fegupol diamond polishing paste (Schmitz-Metallographie, Herzogenrath, Germany).

Material 3 was fabricated from blocks using the CAD/CAM technique. The surface of the material was glazed with Ivocolor (Ivoclar Vivadent AG, Schaan, Liechtenstein).

Material 4 was delivered by the manufacturer as blocks. From these blocks, discs were cut using a trepan burr (Hager & Meisinger GmbH, Neuss, Germany) with a speed of 10.000 rpm under water cooling. The disc surface was polished with a VITA ENAMIC Polishing Set technical/Two Step Polishing System (Vita, Bad Säckingen, Germany).

Material 5 was completely prefabricated by the manufacturer as ready for use in discs. Additionally, the surface was polished using standard NEM polishing materials as described by the manufacturer.

Enamel discs were cut from extracted teeth using a trepan burr (Hager & Meisinger GmbH, Neuss, Germany) with a speed of 10.000 rpm under water cooling. As this was a natural material and the surface should be original, no polishing was performed.

### Electron microscopy (SEM)

For SEM analysis, the biofilms were fixed in 2.5% glutaraldehyde containing 1% polyvinylpyrolidon. They were processed for extracellular matrix presentation following SEM preparation according to standard protocols. The biofilms were subjected to SEM with a Zeiss Sigma VP scanning electron microscope (Zeiss, Oberkochen, Germany) at an acceleration voltage of 1.5 kV using an in-lens and SE detector.

### Surface roughness determination

The surface roughness of human enamel and different materials was determined before the investigation using a high-resolution three-dimensional optical surface measurement device (Infinite Focus G3, Alicona Imaging GmbH, Grambach, Austria). The roughness was measured within three randomly selected areas of uniform sizes (50 × 50 m), resulting in 15 measurements per surface. The mean area roughness (Sa) value was expressed in nm.

### Microbiology

In addition to SEM analysis of microbiological biofilms on materials, the materials were analyzed for bacterial growth. Probes were transferred to sterile saline (0.9% NaCl, Roth, Karlsruhe, Germany) in a volume of 500 μl and vortexed intensively but cautiously for up to 10 min; thus, microorganisms were released from the surface. Aliquots were plated onto Columbia blood agar (Oxoid, Munich, Germany), and the plates were incubated at 36 °C for 24 h to 72 h. Bacterial growth was determined by measuring colony-forming units on the plates. Morphologically different microorganisms were subcultured into pure isolates. For species identification, the biotyping technique (Microflex LT mass spectrometer, Bruker Daltonik, Germany) was used. A single colony was spotted directly on the target and overlaid with 1 μl of matrix solution, followed by air-drying. The loaded plate was then applied to the instrument according to the manufacturer’s instructions. The spectrum of each isolate was compared with those in the database.

### Statistical analysis

The mean value of all roughness measurements on the same disc was used for further statistical evaluation. Prior to the analytical statistics, the data were tested for normality using the Shapiro-Wilk and Kolmogorov-Smirnov tests. As both tests indicated a normal distribution of the data, parametric tests were used for inductive statistics. For comparison of the surface roughness on the different materials, one-way ANOVA with Tukey’s multiple comparisons post hoc test was used. The data distribution is expressed as boxplot graphics, and the statistical study results are presented as tables. Graph Pad Prism Ver. 8 (GraphPad Software, San Diego, CA, USA) was used as the statistical analysis software.

## Results

### Surface roughness determination

**Fig. 3:**
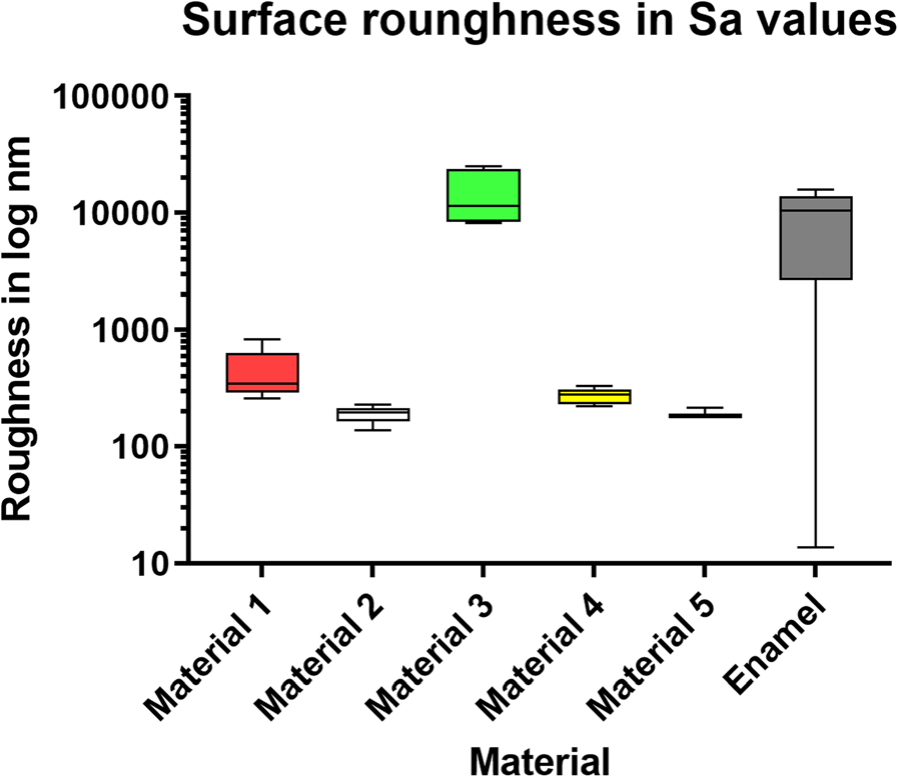
Boxplot graph of the descriptive statistics of the roughness measurements indicating the data distribution of the measurement results as minimum (lower bar), 50% percentile (box), median (line within the box) and maximum (upper bar)

Significant differences were found in the Sa values of the surface roughness between materials 1–3 and 5 and enamel. Material 4 showed no significantly different Sa values. The comparison of material 4 with material 5 also revealed significant differences. All statistical results are summarized in Table 2 and Fig. 3.

**Table 2:** Summary of the statistical results of the surface roughness measurements

	Material 1	Material 2	Material 3	Material 4	Material 5
Material 2	*0.0043*				
Material 3	*< 0.0001*	*0.101*			
Material 4	*0.0879*	*0.8045*	*0.8159*		
Material 5	*0.0045*	*> 0.9999*	*0.0965*	*0.0058*	
Control	*< 0.0001*	*0.0824*	*0.0786*	*0.0046*	*> 0.9999*

*P*-values, ANOVA test for multiple comparisons

### Scanning Electron microscopy (SEM)

#### Material 1

**Fig. 4:**
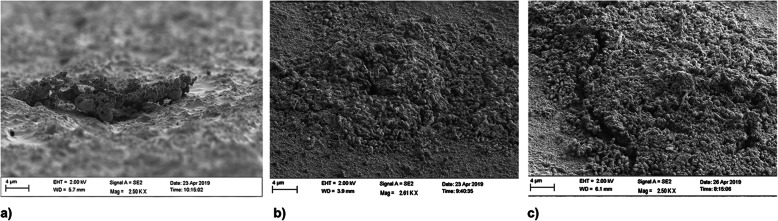
SEM images of biofilm growth on material 1 after 3 h (**a**), 24 h (**b**) and 72 h (**c**)

Three hours after having the volunteers wear the dental splints, a thick irregularly acquired pellicle with scattered nests of organic material and some isolated bacteria was observed (Fig. 4a). Twenty-four hours after biofilm growth, isolated nests of bacterial biofilms were found (Fig. 4b), and after 72 h, an encased thick biofilm covered the material surface (Fig. 4c).

#### Material 2

**Fig. 5:**
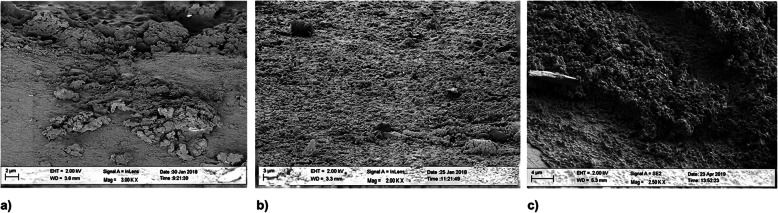
SEM images of biofilm growth on material 2 after 3 h (**a**), 24 h (**b**) and 72 h (**c**)

Three hours after having the volunteers wear the dental splints, a proper pellicle of organic material and some isolated bacteria were observed on the material surface (Fig. 5a). The formed pellicle at 24 h was more robust and thicker than that at 3 h. An encased layer of bacteria (Fig. 5b) covered the whole surface. After 72 h, a thick multilayered mature biofilm was observed on the material surface, supported by a robust organic matrix (Fig. 5c).

#### Material 3

**Fig. 6:**
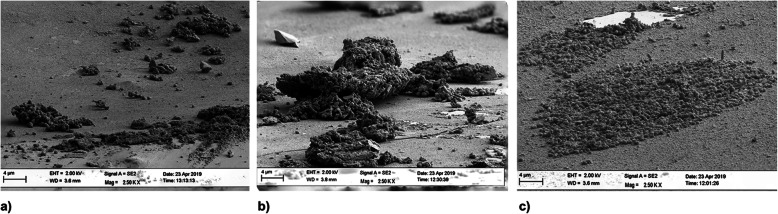
SEM images of biofilm growth on material 3 after 3 h (**a**), 24 h (**b**) and 72 h (**c**)

Three hours after having the volunteers wear the dental splints, an encased thin pellicle was detected, with isolated bacterial cells (Fig. 6a). After 24 h, bacterial nests were found on the surface next to a thick pellicle, but no encased biofilm was observed (Fig. 6b). After 72 h, a thin biofilm nest was detected that did not cover the whole surface (Fig. 6c).

#### Material 4

**Fig. 7:**
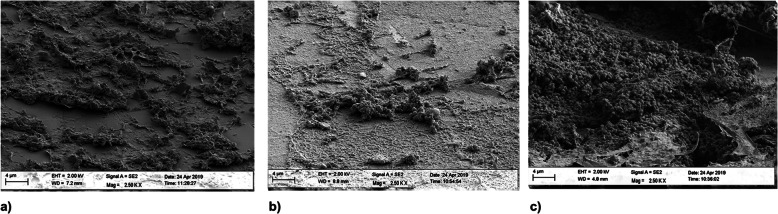
SEM images of biofilm growth on material 4 after 3 h (**a**), 24 h (**b**) and 72 h (**c**)

Three hours after having the volunteers wear the dental splints, a thin encased acquired pellicle with scattered nests of organic matrix was found on the material surface (Fig. 7a). After 24 h, the surface of the acquired pellicle was covered by an organic matrix with some bacterial nests (Fig. 7b). After 72 h, a thick and mature biofilm covered the material surface with embedded rods (Fig. 7c).

#### Material 5

**Fig. 8:**
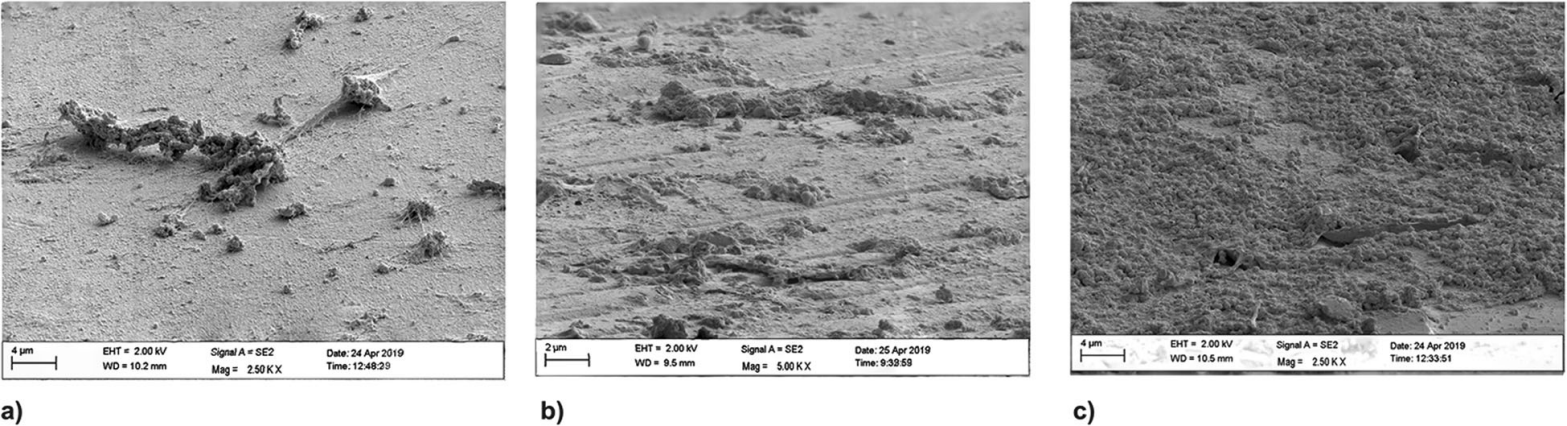
SEM images of biofilm growth on material 5 after 3 h (**a**), 24 h (**b**) and 72 h (**c**)

Three hours after having the volunteers wear the dental splints, a thin, homogeneous acquired pellicle with only a few nests of organic material was found (Fig. 8a). After 24 h, the nests proliferated, and few single bacteria were detected on the surface. The acquired pellicle appeared rather thin (Fig. 8b). After 72 h, the surface was partially covered by a thin monolayer bacterial film with some embedded rods (Fig. 8 c).

#### Enamel

**Fig. 9:**
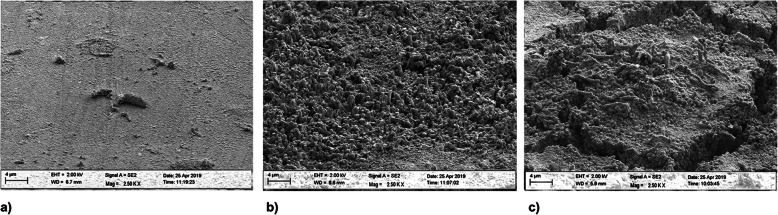
SEM images of biofilm growth on human enamel after 3 h (**a**), 24 h (**b**) and 72 h (**c**)

Three hours after having the volunteers wear the dental splints, an encased pellicle with isolated bacterial nests was detected on the enamel surface (Fig. 9a). After 24 h, a thick biofilm covered the whole surface (Fig. 9b), and the biofilm grew further and became robust and thick after 72 h. Numerous rods were embedded into the biofilm (Fig. 9c).

### Microbiology

Various but typical microbial species that grew on the different material surfaces were isolated and differentiated. Bacteria were generally identified as species belonging to the physiological oral flora. Differences in bacterial colonization among all materials were found after 3 h and 24 h of biofilm formation. After 72 h, the biofilms seemed to be mature. Isolates were composed of mainly *Streptococcus oralis*. The results of the microbiological identification are summarized in Table 3.

**Table 3:** Identified bacteria in the biofilms of the various materials

Material 1	Identified Isolates	Material 2	Identified Isolates	Material 3	Identified Isolates	Material 4	Identified Isolates	Material 5	Identified Isolates
Material (3 h)	Growth, but no identification possible	Material (3 h)	*S.* spp.	Material (3 h)	No bacterial growth	Material (3 h)	Growth, but no identification possible	Material (3 h)	*Rothia dentocariosa* *S. pneumoniae* *N. macacae*
Enamel (3 h)	*S. oralis* *S. mitis*	Enamel (3 h)	*Paenibacillus glucanolyticus*	Enamel (3 h)	*S. mitis* *S. pneumoniae* *Rothia mucilaginosa*	Enamel (3 h)	*Paenibacillus glucanolyticus* *Micrococcus luteus*	Enamel (3 h)	*S. mitis* *S. pneumoniae Rothia mucilaginosa*
Material (24 h)	*S. mitis*	Material (24 h)	Growth, but no identification possible	Material (24 h)	*Rothia dentocariosa* *N. mucosa* *N. flavescens*	Material (24 h)	*S. oralis*	Material (24 h)	Growth, but no identification possible
Enamel (24 h)	*S. oralis* *S. mitis*	Enamel (24 h)	*S. oralis*	Enamel (24 h)	*Streptococcus peroris* *Rothia mucilaginosa*	Enamel (24 h)	Growth, but no identification possible	Enamel (24 h)	*S. peroris* *Rothia mucilaginosa*
Material (72 h)	*S. oralis*	Material (72 h)	*N. macacae* *N. mucosa*	Material (72 h)	Growth, but no identification possible	Material (72 h)	*S. parasanguinis*	Material (72 h)	*S. oralis*
Enamel (72 h)	*S. oralis* *S. mitis*	Enamel (72 h)	*S. periois* *S. infantis* *S. mitis* *N. subflava* *Enterobacter* spp.	Enamel (72 h)	*S. oralis*	Enamel (72 h)	*S. oralis*	Enamel (72 h)	*S. oralis*

S. *Streptococcus,* N. *Neisseria*

## Discussion

Bioadherance is the primary force in the development of biofilms on all natural and artificial surfaces [15, 16, 22–24]. Based on previous studies, it can be concluded that oral biofilm formation occurs in three steps: 1) acquired pellicle formation, 2) pioneer bacterial colonization (nonmature biofilm formation) and finally 3) secondary colonization of various other bacteria (final mature biofilm formation) [1, 22, 23, 25, 26].

A prerequisite for biofilm formation on various surfaces of the oral cavity is the formation of an acquired pellicle [23]. A pellicle is a proteinaceous layer on the material surface that is formed within seconds after cleaning [23]. Protein construction and thickness of the acquired pellicle seem to be dependent on the underlying material [16]. This study demonstrated specific morphological differences of the acquired pellicle on the surface of various dental materials. The composition of the acquired pellicle depends on several surface properties, such as surface energy, surface roughness and material composition [15, 23, 26, 27]. A number of studies have shown that surface roughness is the key factor for the deposition of acquired pellicle and plaque development [26, 28]. The higher the surface roughness is, the better the bacterial adhesion [27, 29]. Increasing roughness enlarges the surface area for bacterial attachment. Our findings showed that biofilm formation occurred rapidly on the enamel and composite. This finding is in concordance with the results of previous studies, which showed that composites and enamel have relatively higher susceptibility to biofilm development [22, 23]. Composite and enamel have a low surface energy and are highly susceptible to biofilm development [23]. The results for the ceramic materials were controversial. A recent study demonstrated that a thin biofilm with a high vitality value developed on dental ceramics [2]. In the present study, two ceramic materials showed fast development of a thick acquired pellicle with consequent growth of a bacterial biofilm, whereas on one ceramic material, only a thin acquired pellicle with a moderate biofilm was formed. This result confirms the assumption that not only the acquired pellicle but also the material composition has an influence on biofilm formation [16, 30]. Metals are generally less susceptible to the development of an acquired pellicle following biofilm development due to their specific surface charge and surface energy [31]. There are differences in bacterial colonization on various metal and metal alloy surfaces, depending on their composition [30, 31]. In this study, the metal alloy (CoCrMo) demonstrated the thinnest acquired pellicle, with moderate bacterial biofilm growth. It has been discussed that the acquired pellicle masks the surface properties of the underlying material to a certain extent [15, 23, 24, 26].

Pioneer colonizer microorganisms adhere to the acquired pellicle, creating a basic biofilm or a nonmature biofilm [24, 31], and vary depending on the environment and the materials to which they adhere [23, 31]. Contradictory results have been published about the composition of microbial pioneer colonizers. Some studies reported that streptococci detected in the oral cavity are always pioneer colonizers [24, 32], while others identified other bacterial species as pioneer colonizers, each depending on the material composition [2, 15, 26, 31]. Pioneer colonizers in oral biofilms have been identified as *Streptococcus sanguinis, S. oralis, S. gordonii, S. mitis, S. mutans, S. sobrinus, Actinomyces naeslundii* and *Capnocytophaga ochracea* [33].

After pioneer colonizers, secondary colonizers, consisting of various species depending on the bacterial composition of the environment, follow; these species can be used predict the bacterial composition of the mature biofilm [1, 24–26]. The mature stage of the oral biofilm usually occurs after 3–5 days and is characterized by thickness and microbial composition [33]. Mature biofilms have high microbiological variability and contain mainly rods that are absent in growing biofilms [24, 26]. In this study, the main bacteria in the mature biofilms on most materials were identified as *Streptococcus oralis*. The differences in the present study were found in the maturation of biofilms on the tested dental materials. The composite material used in this study demonstrated rapid biofilm formation similar the biofilm on enamel. This finding is in concordance with the results of previous studies, which showed that composites and enamel are susceptible to biofilm development and biofilm thickness [23, 34]. The results varied with ceramic materials, which may be due to surface roughness and material composition. In the present study, after 72 h, the metallic material demonstrated a monolayer biofilm with only some rods, which might indicate a nonmature biofilm. Many viable bacteria are found on cobalt-chrome alloys [33]. The oral cavity harbors vast amounts of various bacterial strains [35, 36], and it seems reasonable that the biofilm composition on various natural and artificial surfaces in the oral cavity varies.

This study has some limitations, which are that it is mainly descriptive and no quantification of the different bacteria could be made. Further studies need to be performed with statistical methods to verify the morphologic findings.

## Conclusions

Our morphologic results indicate that within 72 h, mature oral biofilms formed on enamel and various dental materials, except for the surfaces of the metallic and one ceramic material on which, during this time, a thin nonmature biofilm was formed. Oral biofilms depend on the bacterial composition of the host oral cavity, while biofilm maturation on specific restorative materials is influenced by surface properties and material composition.

## Data Availability

All data are available from the corresponding author upon request.

## Abbreviations

SEM: Scanning electron microscopy
Sa: Mean surface roughness
NaCl: Sodium chloride
